# Efficiency analysis by training sequence of high-fidelity simulation-based neonatal resuscitation program (NRP)

**DOI:** 10.1371/journal.pone.0281515

**Published:** 2023-02-10

**Authors:** Seoheui Choi, Hoyeon Shim, Jang H. Lee

**Affiliations:** 1 Department of Paediatrics, Ajou University School of Medicine, Suwon, South Korea; 2 Department of Simulation Center, Hallym University Dongtan Sacred Heart Hospital, Hwaseong-si, South Korea; University of Oxford, UNITED KINGDOM

## Abstract

**Objective:**

This study was conducted to find a more effective education method in a neonatal resuscitation program (NRP) using a high-fidelity simulation that has recently shown positive results in terms of its effectiveness.

**Study design:**

A single-blind prospective cohort study was performed. The high-fidelity simulation model was used in a formal NRP training course for trainees caring for neonatal patients. The trainees were divided into a group that conducted the scenario after the lecture (Group A) and a group that attended the lecture after the scenario (Group B) and they both took the test before, during, and after the training.

**Results:**

The increase in score after theory training was statistically significant in both groups, but the final score did not differ between the two groups. However, when compared by career, in Group A, trainees under 24 months tended to be more effective, and in Group B, trainees over 24 months tended to be more effective.

**Conclusion:**

The difference in short-term memory of trainees according to the order of education identified by the test score was not prominent, but it was found that the degree of difference in test scores for the order of education tended to be different according to the career. It is thought that the effectiveness of the training might be increased by changing the education order according to the degree of experience of each trainee. More effective educational methods should be introduced by continuously developing lectures for repeated education of various trainees in the future.

## Introduction

The Neonatal Resuscitation Program (NRP) is a field of cardiopulmonary resuscitation (CPR) designed and published based on improving the prognosis by providing appropriate early management to newborns [1]. This program is revised every 5 years by the American Heart Association (AHA) and the American Academy of Pediatrics (AAP) following an evidence-based review by the International Liaison Committee on Resuscitation (ILCOR) [2]. The patient group receiving the NRP procedure and technique is in a special situation of childbirth, and the number of practitioners performing the technique is limited, making it difficult for trainees to receive new education and learn how to perform the procedure. As several reports have been published that simulation has an educational effect in a limited environment, several studies are being conducted on the effect of applying simulations to NRP [3–6].

There are many reports that NRP training using high-fidelity simulation provides training effectively [3, 4]. However, the acquisition of knowledge and skills through education is maintained only through continuous repetition of education [7, 8], and simulation is a very effective tool for such repetition education [9]. Several methods have been applied to ensure the continued effectiveness of NRP training, including simulation. As part of this, studies have been reported that applied the method of implementing a booster lecture [10, 11], debriefing through video monitoring [12], and rapid cycle deliberate practice [13, 14].

The authors intend to find a teaching method that allows trainees to receive NRP training methods more accurately and effectively through simulation, considering the early providers who need to work immediately after receiving a licence to work in the medical field. This study was conducted to determine which method is more influential: simulation after lecture (Group A: reflects a student environment) and lecture after simulation (Group B: reflects a real working environment) to identify more memorable education methods for NRP training through simulation.

## Materials and methods

### Study population

Thirty-three neonatal staff (19 neonatal intensive care unit (NICU) nurses, 4 nursery nurses, 6 delivery room (DR) nurses, 2 physician-assisted (PA) nurses, 2 paediatric residents) without experience in simulation training through scenarios were included. According to hospital regulations, trainees were randomly assigned to NRP regular training conducted once a year, and the NRP training was conducted through simulation by developing two scenarios. All trainees were informed in advance about the implementation of NRP education, including the simulation practice, and self-directed learning was recommended in advance. All of them signed a consent form prior to training. This study was reviewed by the Hallym University Dongtan Sacred Heart Hospital Institutional Review Board (IRB) (IRB No. 2021-01-018).

## Materials and methods

As a prospective cohort study, this study was conducted in 8 morning and afternoon sessions from March 22, 2021, to March 26, 2021. Each session consisted of 50 minutes of a short, key-summary lecture, 10 minutes of break, and 2 cycles of simulation, each cycle lasting approximately 40 minutes. Simulation scenarios and tests were developed under the supervision of a neonatal subspecialist with over 10 years of experience based on NRP and confirmed by peer review. The scenario was used in common for all participants. The trainees received training with the same content, and the same test questions were administered 3 times (before, during, and after the training). The simulation consisted of briefing-scenario-debriefing, and video monitoring was applied to the debriefing. The teaching method for each session was arbitrarily divided, and Group A conducted a simulation after the lecture and Group B attended the lecture after simulation education. All trainees were informed that this was a routine, once-a-year, neonatal CPR training and selected a date and time that suited their individual working conditions; therefore, they were blinded to the study situation and intent at that time. Prior to the start of the lecture, informed consent was obtained for the purpose of the experiment, the order of the practice, and participation. This study conducted a prospective cohort of interventions and follow-up measures for each group by conducting training for each group over a continuous period time.

**Fig 1:**
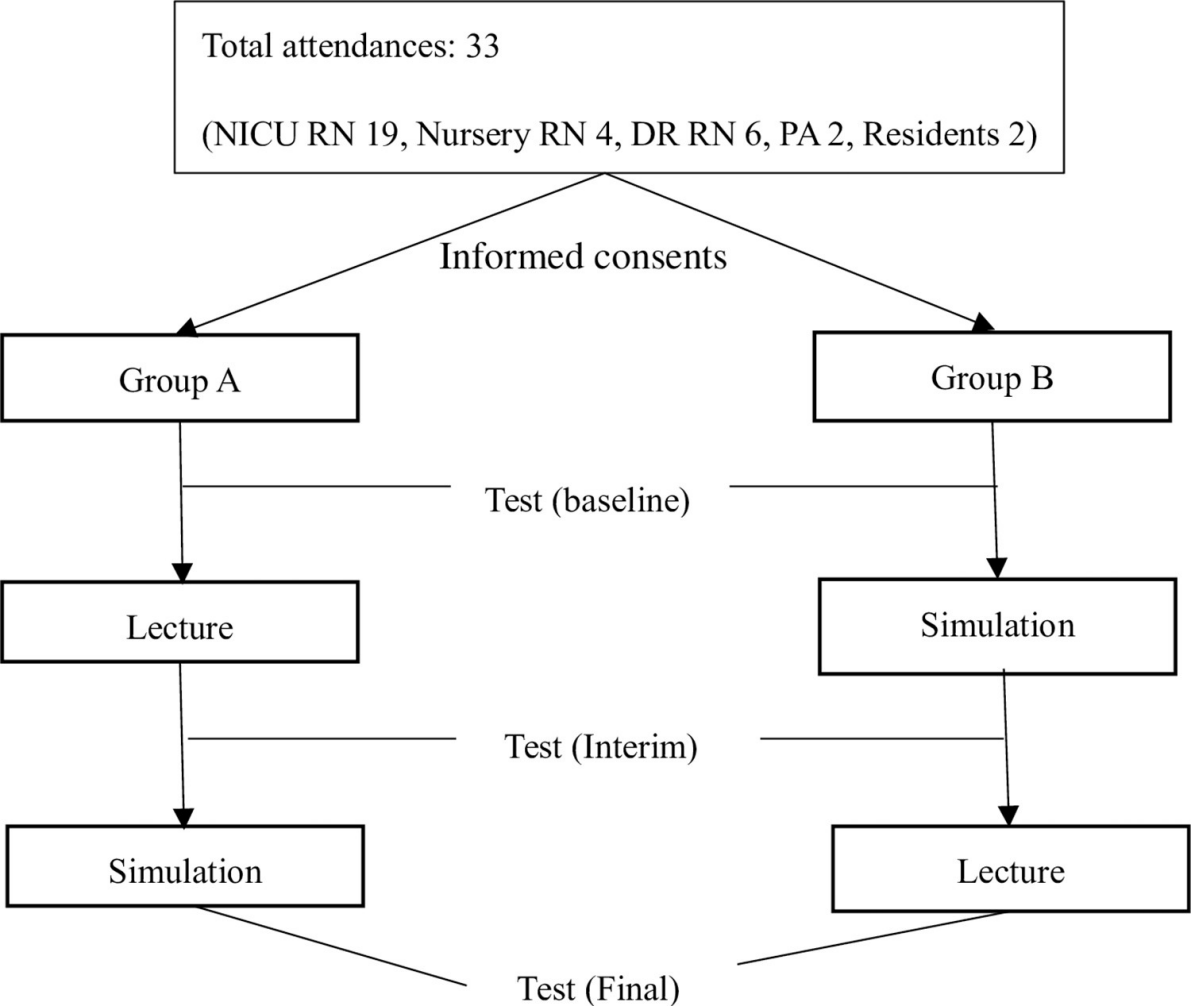
A Schematic diagram of the research.

To increase the effectiveness of the simulation training, the number of participants per session was limited to 5 people. In accordance with the COVID-19 infection prevention rules, participants were quarantined 2 m apart in the classroom, and during the simulation scenario, they wore a mask and practised hand hygiene. In the course of the experiment, the security of the content was emphasized to the subjects each time. The trainee’s test was conducted three times (pre, interim, post) (Fig 1).

Newborn PEDI^®^ Skills Trainer (S109, Gaumard^®^, USA) was used for the simulation model, and Unified Simulator Control Software (UNI^®^ Ink, Control Software for Gaumard^®^ Simulators, Gaumard^®^, USA) was used as a software program for the monitor module system.

### Data analysis

Age, gender, years of career in the relevant department, and 1^st^/2^nd^/3^rd^ test scores were investigated, and the difference in test scores was compared with Student’s *t test* between Group A and Group B to verify the significance with a *p* value <0.05 (SPSS package 21^st^ ed. IBM/SPSS, Chicago, IL). In addition, the difference in test scores was compared between Group A and Group B by subgrouping trainees’ career years before and after 24 months.

## Results

There was no epidemiologic difference between Group A and Group B. The prelecture test scores did not differ between the two groups (32.3±28.4 vs. 37.1±20.4), and interim tests conducted after the first turn showed significant differences between the two groups (65.7±24.0 vs. 47.4±18.2 p = 0.021). It was also confirmed that there was a significant difference between the groups in the difference between the first test score and the second test score (33.4±21.8 vs. 10.29±6.9 p = 0.001). There was also a significant difference between the two groups in the final test score after the second turn (0.63±4.5 vs. 25.29±13.8 p<0.001). Group B had a higher score than Group A, but the difference was not statistically significant (66.3±24.43 vs. 72.7±12.74 p = 0.360). The difference between the first and final test scores was not significantly different between the two groups (34.06±22.6 vs. 35.59±15.7 p = 0.824) (Table 1).

**Table 1:** Demographic findings and total score differentiation.

	Group A (n = 16)	Group B (n = 17)	*P* value
Age (yrs)	27.1±5.2	31.1±9.01	0.127
Gender			
Female	16	17	
Male	0	0	
Position			
NICU+Nursery	13	12	
DR	2	4	
Resident	1	1	
Career (A:B)	20.7±24.2	50.3±73.0	0.130
< 24 months (11:11)	7.85±7.03	11.5±5.7	0.196
≥24 months (5:6)	49.0±24.8	121.3±87.2	0.101
Test Scores			
Preeducation			
Mean	32.3±28.4	37.1±20.4	0.578
Interim-education			
Mean	65.7±24.0	47.4±18.2	0.021
Differentiation	33.4±21.8	10.29±6.9	0.001
Post-education			
Mean	66.31±24.43	72.7±12.74	0.360
Differentiation	0.63±4.5	25.29±13.8	<0.001
Total score differentiation	34.06±22.6	35.59±15.7	0.824

Each group (Group A vs. Group B) was divided into 2 subgroups (the <24 months and the ≥24 months subgroups) according to their experience, and the preliminary test results before the lecture were significantly different in Group B, the ≥24 months experience group (Group A 25.6±25.0 vs. 47.0±32.6 *p* = 0.237, Group B 29.0±20.5 vs. 52.0±9.0 *p* = 0.006). After the first turn, in Group A, the score increase was significantly higher in the <24 months subgroup than in the ≥24 months subgroup (40.6±20.4 vs. 17.6±17.0 *p* = 0.042), and in Group B, the test score was consistently higher in the ≥24 months subgroup, but the increase in score was not statistically significant (12.0±6.9 vs. 7.17±6.2 *p* = 0.166). In the <24 months subgroup, both Group A and Group B showed no statistically significant difference in the score compared to the ≥24 months subgroup, but the difference in scores showed a tendency to increase <24 months (Table 2).

**Table 2:** Subgroup analysis by the order of the training.

	Group A (lecture-Sim) (n = 16)			Group B (Sim-lecture) (n = 17)		
	<24 mon	≥24 mon	*P* value	< 24 mon	≥24 mon	*P* value
	(n = 11)	(n = 5)		(n = 11)	(n = 6)	
Preeducation						
Mean	25.5±25.0	47.0±32.6	0.237	29.0±20.5	52.0±9.0	0.006
Interim-education						
Mean	66.2±23.5	64.6±27.9	0.915	41.0±18.2	59.2±11.9	0.026
Differentiation	40.6±20.4	17.6±17.0	0.042	12.0±6.9	7.17±6.2	0.166
Post-education						
Mean	66.9±24.9	65.0±26.1	0.894	67.7±12.8	81.8±6.3	0.008
Differentiation	0.73±4.9	0.4±3.6	0.885	26.73±16.03	22.67±9.31	0.519
Total score differentiation	41.4±21.9	18.0±15.5	0.33	38.73±18.67	29.8±5.2	0.164

To confirm the learning effect according to their experience, a subgroup analysis was performed targeting those with <24 months and ≥24 months of experience. In the <24 months subgroup, after the first turn, not only were the test scores higher in Group A than in Group B (66.18±23.47 vs. 47.0±18.17 *p* = 0.011) but so were the test score differentiations (40.64±20.39 vs. 12.0±6.87 *p* = 0.001). However, in the test results conducted after the second turn, Group B showed a statistically significant difference in scores between tests (0.73±4.94 vs. 26.73±16.03 p<0.001). The increase in the final score showed a high trend in Group A, but there was no significant difference. In the ≥24 months subgroup, the increase in test score after the second turn in Group B significantly increased (0.40±3.68 vs. 22.67±9.31 p = 0.001), and the increase in the final score showed a high trend in Group B, but there was no significant difference (Table 3).

**Table 3:** Subgroup analysis by careers, 24 months from neonatal care parts.

	<24 mon (n = 22)			≥24 mon (n = 11)		
	A (n = 11)	B (n = 11)	*P* value	A (n = 5)	B (n = 6)	*P* value
Preeducation						
Mean	25.55±25.01	29.0±20.5	0.727	47.0±32.57	52.0±9.03	0.754
Interim-education						
Mean	66.18±23.47	47.00±18.17	0.011	64.6±27.9	59.17±11.87	0.701
Differentiation	40.64±20.39	12.0±6.87	0.001	17.6±17.04	7.17±6.18	0.252
Post-education						
Mean	66.91±24.93	67.73±12.75	0.924	65.0±26.08	81.83±6.27	0.225
Differentiation	0.73±4.94	26.73±16.03	<0.001	0.40±3.68	22.67±9.31	0.001
Total score differentiation	41.36±38.73	38.73±18.67	0.765	18.0±15.5	29.8±5.2	0.167

## Discussion

The lack of NRP clinical experience in neonatal care continues to appear [6, 15]. Kane and Lorant [16] reported that only 45% of graduate general paediatricians were involved in labour. This is related to the shortened training time and decreased opportunities for majors as the number of patients decreases [6]. In this situation, education using simulation is widely used in the medical field, and several papers have been reported that the use of a high-fidelity infant mannequin and that simulation education through scenarios is effective for trainees [3, 17–19].

For the purpose of ongoing efforts to develop more powerful methods for sustaining cognitive abilities and skills, training professionals through simulation is considered safe and effective [20]. It is possible to induce qualitative development through the correction and education of partial behaviours [21]. As part of this effort, this study was conducted to find an appropriate teaching method through high-fidelity simulation.

In this study, no matter which training method was selected, the final test scores after training were similar when compared for the training methods. In particular, Group B showed no significant increase in interim test results, but the final score showed a tendency to be higher than that of Group A. From the interim exam to the final exam, the score of Group A did not increase significantly, while the score of Group B steadily increased, indicating that the score on the final exams was higher. Hakansson et al. [22] reported an increase in brain-derived neurotrophic factor (BDNF) levels when physical exercise was performed for 35 minutes, suggesting that BCNF responsiveness between BDNF levels and the association between cognitive function after an intervention may play an important role in cognitive health. Considering these hypotheses, there is a possibility that neurotrophic factors activated through simulation prelearning play a role, thereby enhancing the effect of final memory learning.

Considering that the nurse group with 2–3 years of career occupies the largest proportion compared to others [23], in each group, less than 24 months and more than 24 months of experience were divided into subgroups. In Group A, the test scores after lectures were higher than the initial test scores regardless of the working year, but the score increase of those who had worked for less than 24 months was statistically significantly higher. Those in the ≥24 months career group tended to score higher on the initial exam, while those in the <24 months career group tended to score higher on the final exam. On the other hand, in Group B, those in the ≥24 months career group had higher initial test scores. According to Sweegers and Talamini [24], regular input is a fundamental property of neural stores and applies to higher-level categories that exist between objects or concepts and the environment. As experience increases, similar experiences accumulate in the relevant field, and memory gradually solidifies, so it is possible that their initial test results were higher. Although there were small changes in the interim exam score after the simulation, the changes in the final exam score after the lecture was high, resulting in a statistically significant increase in final test scores. Those in the ≥24 months career group had higher initial evaluation scores, but there was a difference in average scores according to the order after training, suggesting that event-based recall training may be more effective for those who acquired knowledge through experience.

When the training methods were compared by career, it was confirmed that the score difference occurred more in group A in the interim test scores in the <24 months career group. Conversely, in the ≥24 months career group, the difference in the interim and final exam scores of Group B increased significantly. Hilosaka et al. [25] reported that the basal ganglia regulate body movements according to their values, and these movements occur through dopaminergic circuits; it could be limited in short-term memory, but it could also be expressed as long-term memory by the accumulation of stable circuits. This can lead to sensible results with flexible choices such as increasing career length. Recently, several papers have been published on the existence and role of memory engram cells [26, 27] as well as dopamine circuits as a factor involved in short-term memory. In addition, by constructing episodic memories through a series of events by simulations, the educational effect can be increased [28]. Because the ultimate goal of repeated learning is to maintain long-term memory through repetitive spike stimulation at the cellular level, the technique can be performed smoothly when needed in clinical practice [29]. This suggests that different teaching methods should be applied depending on the number of years of experience and the degree of field experience.

The limitations of this study are that the study was conducted with a small number of volunteers and that only short-term memory was evaluated, with no follow-up of mid-/long-term memory. In this regard, further research is needed.

This study was conducted to identify more effective NRP training methods using high-fidelity simulation. The educational effects of the participants were confirmed through the test scores, and as a result, it is thought that the effect might be increased by changing the education order according to the degree of experience of each trainee. Further research might be needed to verify these findings.

## Supporting information

S1 File (XLSX)
